# Predicting mammographic density with linear ultrasound transducers

**DOI:** 10.1186/s40001-023-01327-9

**Published:** 2023-09-28

**Authors:** Annika Behrens, Peter A. Fasching, Eva Schwenke, Paul Gass, Lothar Häberle, Felix Heindl, Katharina Heusinger, Laura Lotz, Hannah Lubrich, Caroline Preuß, Michael O. Schneider, Rüdiger Schulz-Wendtland, Florian M. Stumpfe, Michael Uder, Marius Wunderle, Anna L. Zahn, Carolin C. Hack, Matthias W. Beckmann, Julius Emons

**Affiliations:** 1 Department of Gynecology and Obstetrics, Erlangen University Hospital, University Breast Center for Franconia, Comprehensive Cancer Center European Metropolitan Area Nuremberg (CCC ER-EMN), Friedrich-Alexander University Erlangen-Nuremberg, Universitätsstrasse 21-23, 91054 Erlangen, Germany; 2 Biostatistics Unit, Department of Gynecology and Obstetrics, Comprehensive Cancer Center Erlangen-EMN, Friedrich-Alexander University Erlangen-Nuremberg, Erlangen, Germany; 3 Department of Radiology, Erlangen University Hospital, Friedrich-Alexander University Erlangen-Nuremberg, Erlangen, Germany

**Keywords:** Percent mammographic density, Ultrasound, Breast cancer risk

## Abstract

**Background:**

High mammographic density (MD) is a risk factor for the development of breast cancer (BC). Changes in MD are influenced by multiple factors such as age, BMI, number of full-term pregnancies and lactating periods. To learn more about MD, it is important to establish non-radiation-based, alternative examination methods to mammography such as ultrasound assessments.

**Methods:**

We analyzed data from 168 patients who underwent standard-of-care mammography and performed additional ultrasound assessment of the breast using a high-frequency (12 MHz) linear probe of the VOLUSON^®^ 730 Expert system (GE Medical Systems Kretztechnik GmbH & Co OHG, Austria). Gray level bins were calculated from ultrasound images to characterize mammographic density. Percentage mammographic density (PMD) was predicted by gray level bins using various regression models.

**Results:**

Gray level bins and PMD correlated to a certain extent. Spearman’s ρ ranged from − 0.18 to 0.32. The random forest model turned out to be the most accurate prediction model (cross-validated *R*^2^, 0.255). Overall, ultrasound images from the VOLUSON^®^ 730 Expert device in this study showed limited predictive power for PMD when correlated with the corresponding mammograms.

**Conclusions:**

In our present work, no reliable prediction of PMD using ultrasound imaging could be observed. As previous studies showed a reasonable correlation, predictive power seems to be highly dependent on the device used. Identifying feasible non-radiation imaging methods of the breast and their predictive power remains an important topic and warrants further evaluation.

*Trial registration* 325-19 B (Ethics Committee of the medical faculty at Friedrich Alexander University of Erlangen-Nuremberg, Erlangen, Germany).

## Background

Mammographic density (MD) is defined as the proportion of the area of dense regions on a mammogram to the whole area of the breast. Percentage mammographic density (PMD) reflects breast tissue composition, with dense areas appearing lighter than non-dense areas [1, 2]. Women with a high PMD have a higher risk for developing breast cancer (BC) [3–8]. A case–control study showed high correlation of the absolute dense area (DA) and PMD [7]. The DA was furthermore identified as a BC risk factor [9]. In the context of BC diagnostics, high MD was positively associated with potential masking of BC [10]. No difference in survival between interval cancers and screen-detected cancers for high breast density [11]. Moreover, no association of high MD with risk of death from breast cancer was observed [12]. A recent retrospective analysis added to these results, as no association between PMD and overall survival (OS) was observed despite MD being one of the strongest risk factors for BC [13].

Changes in PMD occur dynamically over the course of a lifetime, the causes of which appear to be multifactorial. MD and age are inversely related: while PMD is higher in premenopausal women, postmenopausal women have significantly lower breast density [14, 15]. In several previous studies, average PMD was not only inversely associated with age, but also with body mass index (BMI) [4, 16–19].

Changes in breast tissue density are also observed in women exposed to exogenous hormonal influence. While hormone replacement therapy (HRT) is associated with higher density scores [20–24], endocrine treatment with tamoxifen or an aromatase inhibitor can reduce breast density [25, 26].

MD is inversely associated with parity [27–30]. Studies have shown that PMD declines with a higher number of pregnancies as well as with a younger age at the first birth [31, 32]. Within about 2 years after the first full-term pregnancy, an average loss of PMD by 12% can be observed [30]. A previous retrospective study assessed the association between the number of full-term pregnancies and PMD relative to age and BMI and observed an inverse correlation between PMD and the number of full-term pregnancies in patients older than 45 years, but not in patients younger than 45 years [31]. Breastfeeding is also associated with reduced breast density [32]. Data from a large analysis of epidemiological studies showed that the relative risk for BC declined by 4.5% per 12 months of breastfeeding [33]*.* These reproductive factors have been shown to reduce the risk for BC while low parity on the other hand is a considerable risk factor for BC [34]. Considering these factors, it might be possible that a reason for lower BC risk is, in fact, the decline in PMD.

Mammography is the standard-of-care method of imaging in BC screening, presenting a reproducible method which is applied at pre-specified intervals [35]. Mammography screening contributed to a decrease in mortality rates as well as in higher stage BC in Germany [36]. However, there are certain disadvantages to this method, creating the need for alternative assessment tools. While radiation doses in mammography have decreased over the last decades, exposure to such ionizing radiation, especially from repeated mammography may lead to radiation-induced BC [35, 37]. Mammography is, therefore, of limited eligibility outside of routine screening programs, especially with regard to younger women. One image-based approach to measure breast density without the use of ionizing radiation is the assessment via MRI. Studies showed reasonable correlation of MRI with PMD [38–42]. While MRI-based methods may allow reasonable prediction of MD, they are expensive and of limited availability.

To learn more about PMD and possible informative value about BC risk, it is important to establish other examination methods. In a previous single-center study, we could demonstrate that B-mode ultrasound imaging was associated with PMD in women who underwent routine mammography [6]. Assessing breast density via ultrasound appears to be a time- and cost-efficient method that can be carried out repeatedly on young and/or pregnant women. However, up to date, there is no widely established and validated method for predicting PMD using ultrasound images. Ultrasound systems from different manufacturers could yield different results as image processing and the available formats can vary [43].

The aim of the present work was to assess the correlation of PMD and ultrasound imaging of the breast using a high-frequency (12 MHz) linear ultrasound probe and the VOLUSON^®^ 730 Expert system (GE Medical Systems Kretztechnik GmbH & Co OHG, Austria) for the prediction of PMD.

## Methods

### Patients

**Fig. 1:**
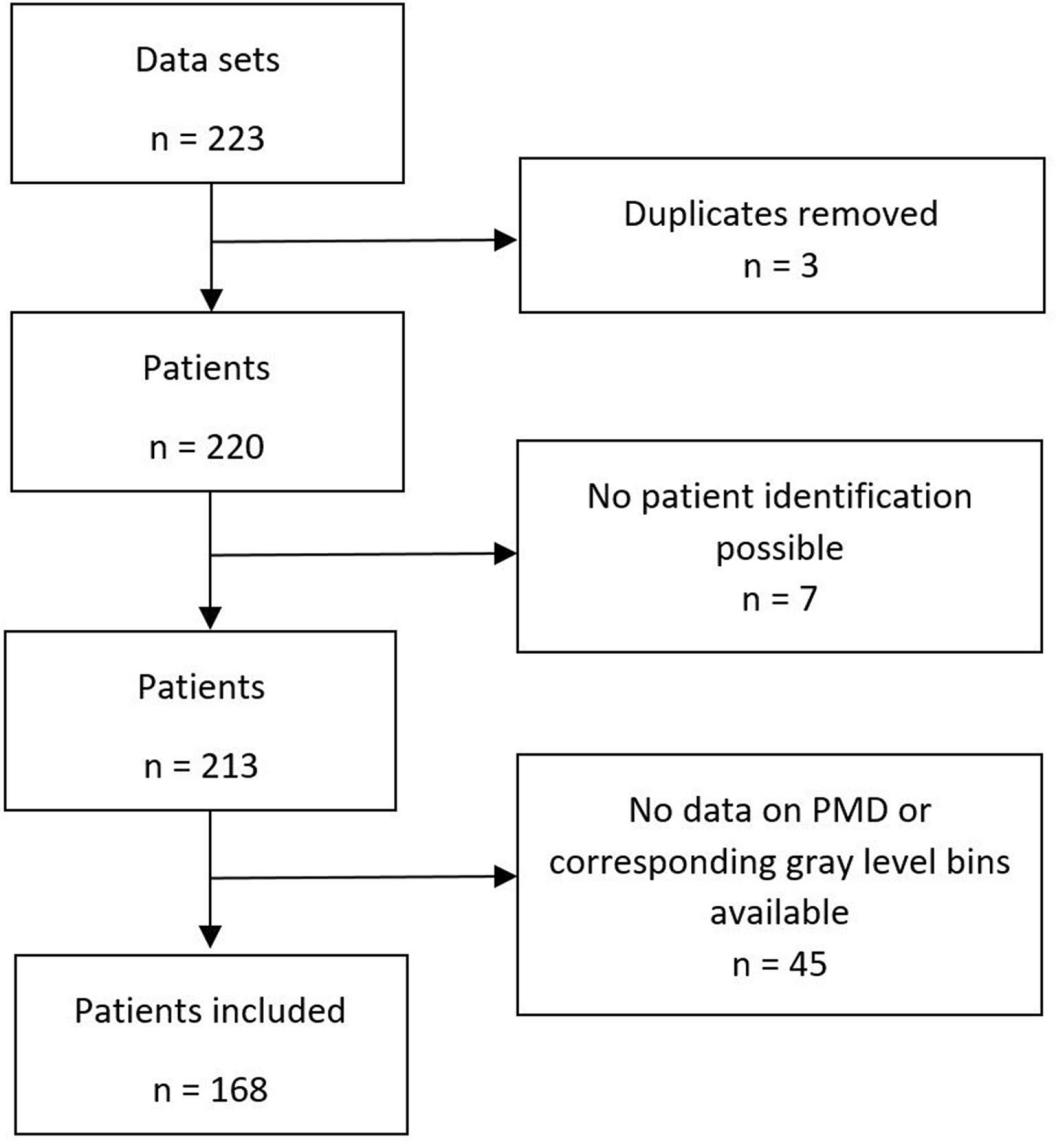
Flowchart of patient selection

Between January 2014 until October 2018, patients were recruited as part of the iMODE-B study (imaging and molecular detection of breast cancer). Imaging and data were retrieved at the University Breast Center for Franconia, at the University of Erlangen–Nuremberg, Germany. Participating patients that had received in-house standard-of-care-mammograms received an additional ultrasound imaging of the breast. Mammography was performed for reasons such as routine or intensified screening, current malignancy or suspicious lesions of the breast, or a history of BC. Patients were eligible for analysis if mammography of the healthy breast was available (BI-RADS 3 or lower) and time between mammography and breast ultrasound was less than 3 months. 168 patients were included in the final analysis. Refer to Fig. 1 for detailed information on the patient selection process.

### Patient recruitment was performed between January 2014 and October 2018

The study was approved by the Ethics Committee of the medical faculty at Friedrich Alexander University of Erlangen-Nuremberg, Erlangen, Germany and was conducted under the 1975 Declaration of Helsinki. All patients provided written informed consent.

### Data acquisition

All patient and tumor characteristics were documented conforming to the requirements of the German Cancer Society (Deutsche Krebsgesellschaft) and the German Society for Breast Diseases (Deutsche Gesellschaft für Senologie) as part of certification processes [44]. Additional clinical data was acquired as part of in-house routine anamnesis.

### Ultrasound imaging

Ultrasound imaging of the breast was performed as described in our previous work [6]. 5 ultrasound images were obtained per breast, one above the mammilla and one of each quadrant of one breast without breast lesions. We used a high-frequency (12 MHz) linear probe of the VOLUSON^®^ 730 Expert system (GE Medical Systems Kretztechnik GmbH & Co OHG, Austria). Images were digitally stored as eight-bit gray scale files. Pictograms were annotated to the images to identify each quadrant, respectively. To avoid misinterpretation and over- or underestimating of PMD, the relevant breast tissue was annotated by two investigators independently. The breast tissue between the muscle and the skin was defined as the region of interest (ROI). The biggest rectangular space possible in the ROI was selected for further assessment. Measurement results from all five images were combined by summation, leading to a single, combined ROI per breast.

From these images, a file in the.xml-format was generated and gray level histograms were extracted. The distribution of gray scales was assessed automatically and provided the number or percentage of pixels within the ROI concerning a gray level value (GLV) or a range of GLVs. Gray level histogram features were calculated to characterize MD. Since an image is made up of pixels, it can be represented as a matrix in which each entry is a variable with values from 0 to 255, describing the gray level. This results in 16 Gy level histogram features by equally dividing the full spectrum of all gray levels into 16 categories (“bins”) and determining the percentage frequency of pixels in each bin [45, 46]. Since the sum over all 16 Gy level bins equals 100% by definition, 15 out of 16 Gy level bins contain all information. For that reason, the 16th bin was omitted at the analysis.

### Acquisition of mammographic density

The following methodology was used in several previous works [4, 6, 7, 10, 13, 30, 31, 45, 47–50]. Quantitative computer-based threshold density assessments were carried out by two different readers. To assess the density proportion, the readers used the Madena software program, version 3.26 (Eye Physics, LLC, Los Alamitos, California, USA) [13]. If mammograms and ultrasound images for both breast sides were available and eligible (e.g. without breast lesions), the measurements of a randomly chosen side were used for analysis. Averages of the two observers’ values for percentage mammographic density (PMD) were used for analysis.

### Statistical analysis

The correlations between gray level bins and PMD were calculated using Spearman’s correlation coefficient ρ.

Various regression models with gray level bins as predictors and PMD as the outcome were set up: A null model without any predictors (M0), an ordinary linear regression model with all (i.e., 15) gray level bins (M1), a linear regression model similar to (M1) but with stepwise backward feature selection (M2), a linear regression model with all gray level bins, each as cubic spline function with two degrees of freedom (M3), a linear regression model similar to (M3) but with stepwise backward feature selection (M4), a lasso model (M5), a ridge regression model (M6), and a random forest model (M7).

The prediction performance of the models was assessed using the mean squared error (MSE) and the *R*^2^ statistic. These measures were obtained by 100 times threefold cross-validation [47, 51, 52]. In particular, all model-building steps were performed on training data, and the performance of the model was assessed on validation data that had not been used for model building. The model with the smallest cross-validated MSE was considered as the *final model*. Apparent measures on the complete dataset were calculated to assess overfitting.

In order to illustrate prediction performance of the final model when applied to future patients, the study population was once more randomly divided into a training set (2/3 of the patients), where the final model was fitted, and a validation set (remaining 1/3 of the patients), where the model was applied to. The observed PMD for a patient in the validation set was then plotted against its predicted PMD.

The *R*^2^ statistic is related to the MSE and takes values from 0 to 1 when applied to training data. It may also take values below zero when applied to validation data. A low MSE value implies a high *R*^2^ value.

The calculations were carried out using the R system for statistical computing (version 3.6.1; R Development Core Team, Vienna, Austria, 2019).

## Results

### Patient characteristics

Characteristics of the study population are shown in Table 1. Mean age of the included subjects was 50.1 years, mean BMI was 24.6 kg/m^2^. In total, 141 patients had a history of at least 1 pregnancy, while 23 patients had never been pregnant. 115 subjects indicated that they had breastfed their children, while 40 subjects had never breastfed. 91 patients had a known history of BC and 6 patients had a known history of in situ carcinoma of the breast (DCIS).

**Table 1:** Characteristics of study population

Characteristic	Mean and SD or *N* and %
Age (years)	50.1 (11.6)
BMI (kg/m^2^)	24.6 (4.6)
Gravida	
0	23 (14.0)
1	42 (25.6)
2	52 (31.7)
3+	47 (28.7)
Para	
0	30 (18.0)
1	43 (25.7)
2	67 (40.1)
3+	27 (16.2)
Breastfeeding	
Yes	115 (74.2)
No	40 (25.8)
Oral contraception	
Yes	24 (14.5)
No	142 (85.5)
Hormone replacement therapy	
Yes	1 (0.6)
No	166 (99.4)
Anti-hormone therapy	
Yes	61 (36.3)
No	107 (63.7)
Known self-history of breast cancer	
Yes	91 (54.1)
No	77 (45.9)
Known self-history of in situ-carcinoma of the breast	
Yes	6 (3.6)
No	162 (96.4)

*SD* standard deviation, *BMI* body mass index

### Ultrasound measures and percentage mammographic density

A total of 168 patients were analyzed. Of each of these patients, a combined ROI of 5 ultrasound images could be assessed with regard to PMD correlation of standard-of-care mammography.

**Fig. 2:**
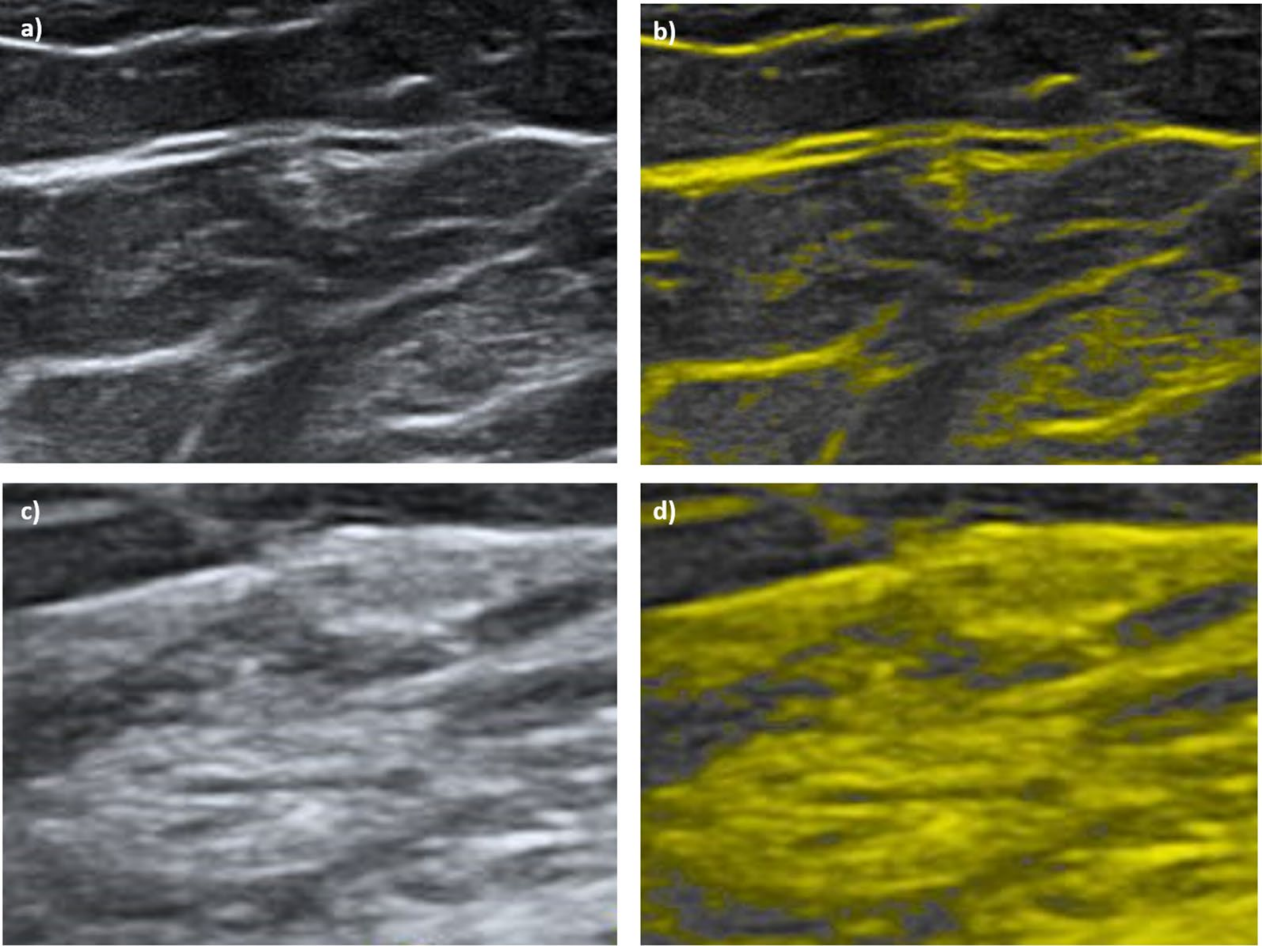
Example of ultrasound images for one patient with low mammographic density (**a**, **b**) and one patient with high mammographic density (**c**, **d**). Dotted lines refer to the pectoralis muscle. Colored markings indicate areas with high breast density. For better visualization, the Madena software was used. **a** The original ultrasound image of the left breast, upper outer quadrant for a patient with low breast density, indicated by only small colored areas in **b**. **c** The original ultrasound image of the left breast, upper outer quadrant for a patient with high breast density indicated by larger colored areas in **d**

Figure 2 shows examples of ultrasound images for a patient with high mammographic density and a patient with low mammographic density.

**Fig. 3:**
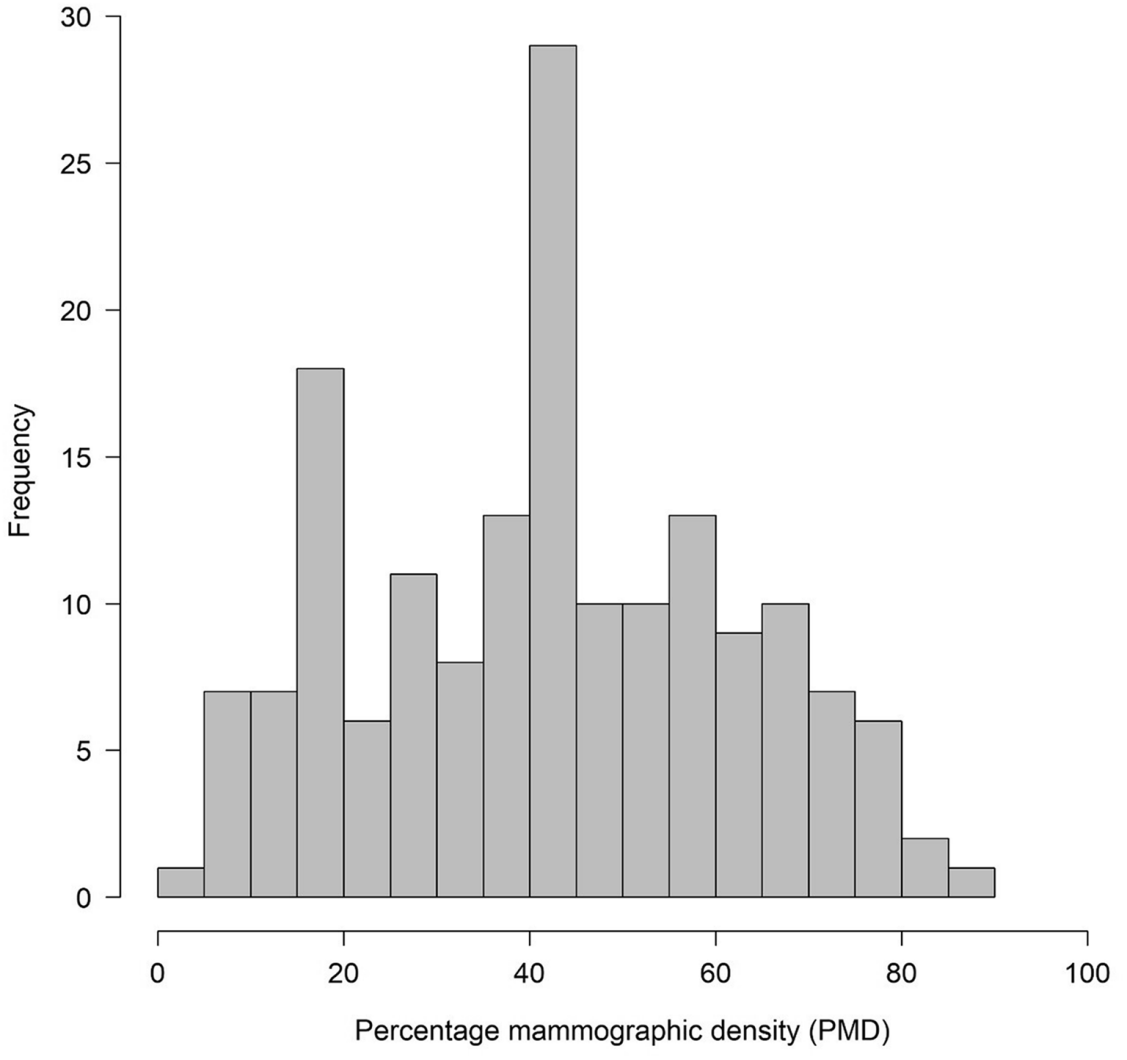
Distribution of percentage mammographic density (PMD)

The analysis of mammograms provided the following results: Mean and median PMD was 42.2% and 43.2%, respectively. PMD was 26.8% or less in 25% of all women, whereas it was 58.5% or greater in another 25% of the women (interquartile range). Figure 3 shows the distribution of PMD in the study population.

### Prediction of PMD with ultrasound features

**Fig. 4:**
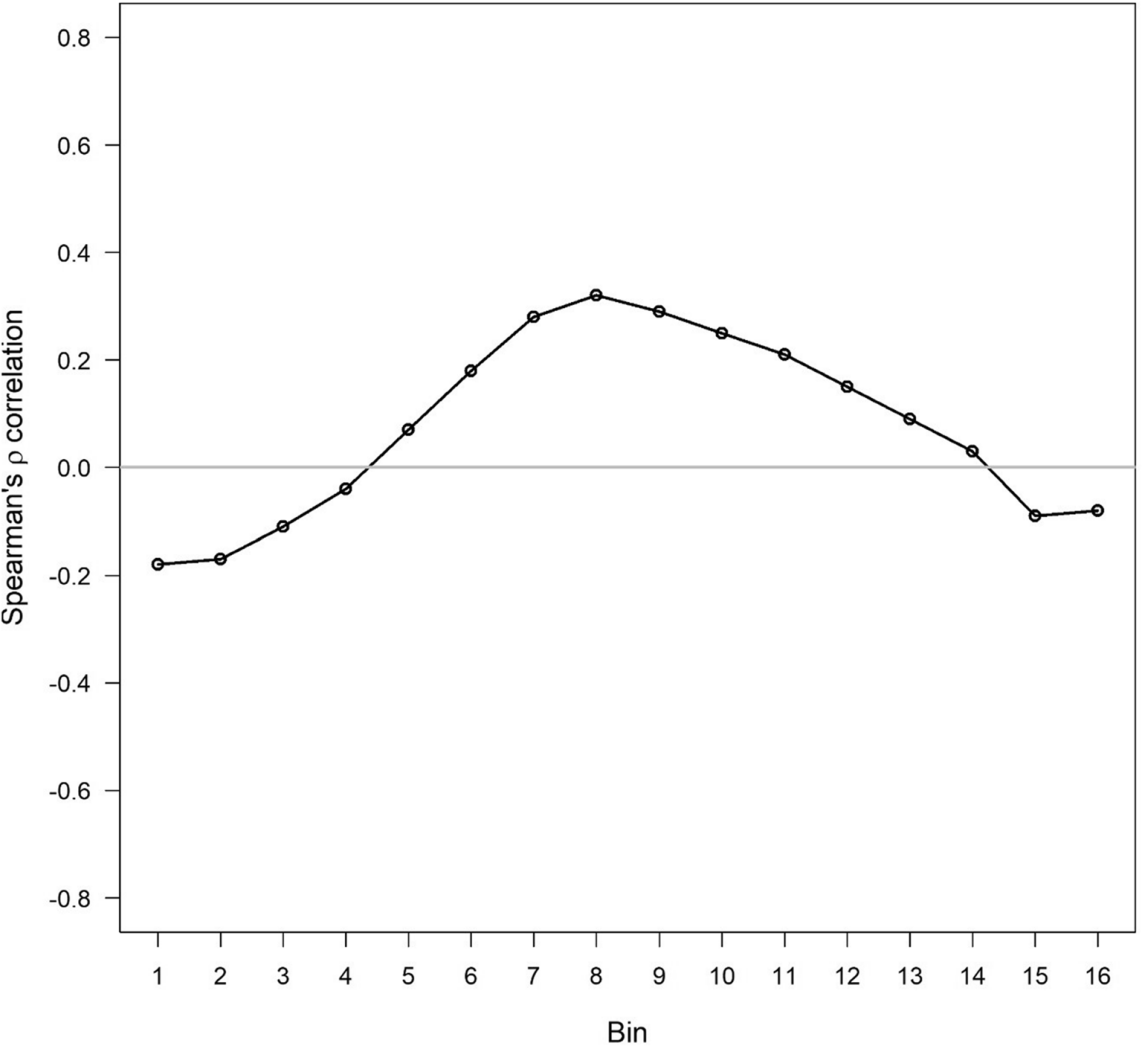
Correlation of gray level bins with percentage mammographic density (PMD). The full spectrum of all gray levels was divided into 16 categories (“bins”) from white (= 1) to black (= 16)

Figure 4 shows the correlation of gray level bins with PMD. In our patient collective, gray level bins and PMD correlated to a certain extent. Spearman’s ρ ranged from − 0.18 to 0.32. The highest positive correlation according to Spearman’s ρ was found for bin 8.

**Fig. 5:**
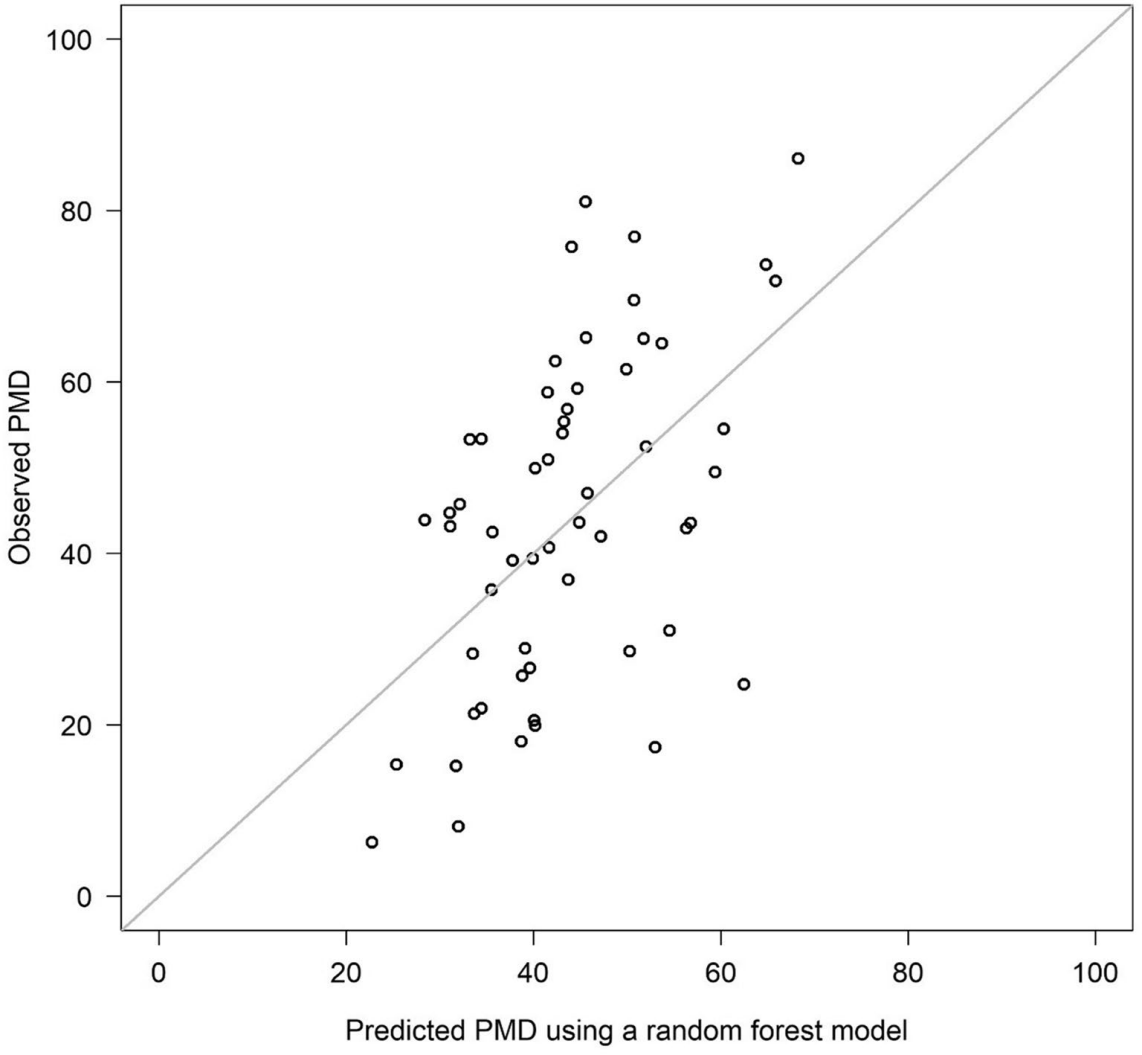
Predicted and observed percentage mammographic density (PMD) values on a validation dataset (one-third of the patients), based on a random forest prediction model fitted on training dataset (two-thirds of the patients)

The performances of the prediction models for PMD are shown in Table 2. The random forest model M7 turned out to be the most accurate prediction model (cross-validated MSE, 0.0291). As expected, the lower the MSE values the higher the *R*^2^ values. M7 had the greatest cross-validated *R*^2^ value (0.255), followed by M4 with a cross-validated *R*^2^ value of 0.114. All other models had values around zero. Figure 5 shows the observed PMD and predictions on a validation dataset using M7 which had previously been fitted on training data. In this example, the *R*^2^ value was 0.28.

**Table 2:** Performance of the prediction models for percentage mammographic density (PMD)

Prediction model	Apparent measures		Cross-validated measures	
	MSE	*R*^2^	MSE	*R*^2^
M0: Null model	0.0396	0.000	0.0400	− 0.021
M1: Linear regression	0.0281	0.289	0.0391	− 0.002
M2: Linear regression with variable selection	0.0285	0.280	0.0386	0.011
M3: Cubic splines	0.0187	0.528	0.0370	0.053
M4: Cubic splines with variable selection	0.0200	0.494	0.0346	0.114
M5: Lasso	0.0294	0.256	0.0367	0.061
M6: Ridge regression	0.0308	0.222	0.0368	0.060
M7: Random forest	0.0049	0.876	0.0291	0.255

*MSE* mean squared error

The apparent performance measures for M7 (MSE, 0.0049; *R*^2^, 0.876; Table 2) were much better than the corresponding cross-validated values, indicating strong overfitting of M7 to the study data.

## Discussion

MD is a significant risk factor for BC and has, therefore, been a much-studied topic with regard to diagnostics and potential prevention. There is profound evidence that women with a high MD have a higher risk for the development of breast BC [3–7]. Moreover, high MD is positively associated with potential masking of BC, potentially resulting in later diagnosis and thus unfavorable prognosis [10].

As MD is closely linked to the number of pregnancies, understanding changes in breast tissue composition during and after pregnancies is of importance [4, 53]. One study assessed changes in the breast volume at the beginning and after a pregnancy using a three-dimensional surface assessment technique [54]. Assessing the correlation of changes in breast volume and changes in MD could be of interest in future trials.

Mammography is used as the standard-of-care method of imaging in BC screening in early stages [35]. While radiation doses in mammography have decreased over the last decades, exposure to such ionizing radiation, especially from repeated mammography may lead to radiation-induced breast cancer [35, 37]. Availability of infrastructure and personnel is limited for mammography. The assessment itself is uncomfortable or even painful for the patients, as the breast is compressed to create reproducibility and enhance imaging quality. To sum up, the usage of mammography is of limited eligibility outside of routine screening programs, especially with regard to younger women.

Several other potential methods of measuring MD have been assessed with somewhat promising results. There are also non-imaging methods such as photo acoustics described in literature [55]. However, these approaches are not based on broadly available imaging techniques and are, therefore, of limited eligibility for clinical routine.

One image-based approach to measure breast density without using ionizing radiation is the assessment via MRI. Studies showed reasonable correlation of MRI with PMD [38–42, 56]. While MRI-based methods to assess breast density may allow reasonable prediction of MD, they have the disadvantage of being somewhat expensive and elaborate and are of limited availability.

Ultrasound imaging of the breast, on the other hand, is a time- and cost-efficient assessment tool. Ultrasound systems are widely available and yield no radiation exposure for the patient. Sonography can be carried out repeatedly and in shorter intervals, depicting a feasible assessment technique for young and/or pregnant women. However, up to date, there is no widely established, validated method for predicting PMD using ultrasound images.

The aim of our previous work was to predict PMD based on sonography and elastography. There, we showed that B-mode images of the normal breast tissue allowed prediction of PMD as assessed via mammography [6]. These results were consistent with other studies [57, 58].

The present work was designed to assess the correlation of PMD and ultrasound imaging of the breast using a high-frequency (12 MHz) linear ultrasound probe in a rather large cohort and examine the predictive value of this imaging assessment method.

Data of 168 patients was evaluated in the course of the present work. Sonography assessment was not fit to support findings of Jud et al. [6]. Ultrasound images showed limited predictive power for PMD when correlated with the corresponding mammograms. *R*^2^ values were consistently low, with the random forest model M7 turning out to be the most accurate prediction model. This effect could be due to heterogeneity in patient characteristics with regard to several parameters. Patients with a history of BC were included as well as patients without current or past malignancies of the breast. As BC could also have an influence on breast tissue density, this indicates heterogeneity for PMD as well as sonography findings, leading to limited eligibility for validation processes. Moreover, patients were eligible for assessment in the current trial regardless of number of pregnancies and time of breastfeeding. These parameters were collected but had no influence on assessment or sub-analyses.

For future trials, a special focus on a less heterogeneous patient collective, especially with regard to breast cancer history could lead to higher *R*^2^ results. In a homogenous collective, different prediction models than the random forest model could turn out to be more accurate.

Ultrasound assessments were carried out by different investigators. In our previous work, automated ultrasound image analysis was performed. Inter-observer differences could potentially inflict the validity of assessment and result in deterioration of predictive power.

Differences in image processing between the used ultrasound machines could have potentially influenced outcome results. For example, the device used in the current work has integrated optimizing presets, which does not apply for the Siemens machine (Acuson Antares premium edition, Siemens, Germany) used in our previous work, which assessed raw data [6]. For future trials, this should be taken into consideration. The use of standardized presets could potentially minimize inter-observer bias caused by individually altered imaging settings. The use of raw data could provide unaltered images, if individual use of settings is avoided.

We assessed gray level bins and correlated these to PMD. However, additional texture features could be analyzed to predict PMD and provide different results in future studies.

Overall, the results allowed no reliable prediction of PMD using ultrasound imaging. These results differ from our previous work, where prediction was considerably stronger (*R*^2^ = 0.67 for B-Mode ultrasound imaging) [6].

## Conclusion

Our results did not align with previous studies. No reliable prediction of PMD using ultrasound imaging was observed. Larger studies are needed to assess ultrasound imaging with regard to prediction of mammographic density and eventually breast cancer risk. Identifying feasible non-radiation imaging methods of the breast and their predictive power in young or pregnant women remains an important topic and warrants further evaluation.

## Data Availability

The datasets used and/or analysed during the current study are available from the corresponding author on reasonable request.

## Abbreviations

BC: Breast cancer
DA: Absolute dense area
DCIS: In situ carcinoma of the breast
GLV: Gray level value
HRT: Hormone replacement therapy
MD: Mammographic density
MSE: Mean squared error
PMD: Percentage mammographic density
ROI: Region of interest
